# Epidemiology of Children and Young People Presenting to Australian Emergency Departments for the Management of Acute Severe Behavioural Disturbance: Secondary Analysis of a Randomised Controlled Trial

**DOI:** 10.1111/jpc.70499

**Published:** 2026-07-17

**Authors:** Elyssia M. Bourke, Kate Klein, Chris J. Selman, Katherine J. Lee, Amit Kochar, Deborah Shellshear, Meredith L. Borland, Shane George, Shefali Jani, Doris Tham, Michael Gordon, Chidambaram Prakash, Andrew Davidson, Catherine L. Wilson, Natalie Phillips, Gaby Nieva, Jonathan C. Knott, Franz E. Babl, Simon S. Craig, the Paediatric Research in Emergency Departments International Collaborative (PREDICT) Network, Hanan ElGharib, Lauren Page, Vivian Truong, Naomi Loftus, Danielle Kallianiotis, Amy Carle, Amanda Williams, Bojana Gladanac, Sharon O'Brien, Patricia Clifford, Melissa O'Brien Smith, Anne‐Maree O'Regan, Lisa Properjohn, Katy Whitten, Catherine Power, Gaby Nieva, Simone Hughes, Lara Caruso, Katie Northcott, Natalie Phillips, Angus Jones, Anne Hinde, Amy Coulson, Nadia Schmidt, Jessica Waghorn, Marietta John‐White, Riku Haataja, Zachary Wilder, Deepali Thosar, Areeba Athar

**Affiliations:** ^1^ Emergency Research Group, Clinical Sciences Murdoch Children's Research Institute Melbourne Victoria Australia; ^2^ Department of Emergency Medicine The Royal Children's Hospital Melbourne Victoria Australia; ^3^ Department of Critical Care The University of Melbourne Melbourne Victoria Australia; ^4^ Department of Paediatrics University of Melbourne Melbourne Victoria Australia; ^5^ Department of Emergency Medicine The Royal Melbourne Hospital Melbourne Victoria Australia; ^6^ Department of Emergency Medicine Ballarat Base Hospital (Grampians Health Services) Ballarat Victoria Australia; ^7^ Clinical Epidemiology and Biostatistics Unit Murdoch Children's Research Institute Melbourne Victoria Australia; ^8^ Melbourne Children's Trial Centre Murdoch Children's Research Institute Melbourne Victoria Australia; ^9^ Department of Emergency Medicine Women and Children's Hospital Adelaide South Australia Australia; ^10^ Department of Acute Care Medicine Adelaide University Adelaide South Australia Australia; ^11^ Department of Emergency Medicine Queensland Children's Hospital Brisbane Queensland Australia; ^12^ Department of Emergency Medicine Perth Children's Hospital Perth Western Australia Australia; ^13^ Division of Emergency Medicine and Paediatrics School of Medicine, University of Western Australia Perth Western Australia Australia; ^14^ Department of Emergency Medicine and Children's Critical Care Gold Coast University Hospital Southport Queensland Australia; ^15^ School of Medicine and Dentistry Griffith University, Southport Queensland Australia; ^16^ Child Health Research Centre The University of Queensland South Brisbane Queensland Australia; ^17^ Department of Emergency Medicine The Children's Hospital at Westmead Sydney New South Wales Australia; ^18^ The Children's Hospital at Westmead Clinical School the University of Sydney Sydney New South Wales Australia; ^19^ Department of Emergency Medicine Western Health (Sunshine Hospital) Melbourne Victoria Australia; ^20^ Department of Psychiatry Monash Medical Centre Melbourne Victoria Australia; ^21^ Department of Psychiatry Royal Children's Centre Melbourne Victoria Australia; ^22^ Department of Anaesthesia Royal Children's Hospital Melbourne Victoria Australia; ^23^ Paediatric Emergency Department Monash Medical Centre Melbourne Victoria Australia; ^24^ Department of Paediatrics School of Clinical Sciences, Monash University Melbourne Victoria Australia

**Keywords:** adolescent behaviour, behavioural problem, child behaviour, disruptive behaviour, dysfunctional behaviour, emotional disturbances, Psychology

## Abstract

**Aim:**

To describe the epidemiological characteristics of children and young people presenting to the emergency department (ED) with acute severe behavioural disturbance (ASBD) who were deemed to require oral sedative medication.

**Methods:**

Secondary analysis of a randomised controlled open‐label multi‐centre trial of oral olanzapine versus oral diazepam for the management of ASBD in children and young people aged nine to 17 years for whom epidemiological data were recorded.

**Results:**

There were 348 participants enrolled in the randomised controlled trial (RCT). The majority were female (215/348, 62%) with a mean age of 14.6 years (standard deviation 2.2). The most common pre‐existing medical or mental health condition was anxiety (122/299, 35%) followed by attention deficit hyperactivity disorder and autism spectrum disorder (33% and 32%, respectively). Two‐thirds of the study population (216/348, 62%) had previously attended the ED for ASBD management and 61% (212/348) reported previous intentional self‐harm. Nearly three‐quarters (247/348, 71%) were accessing psychiatric care in the community prior to their ED presentation. Half of the study population (178/348, 52%) presented to the ED with emergency services (e.g., ambulance, police). The median length of stay in the ED was 5.7 h (interquartile range 3.9–10.2 h) and 28% (98/348) of study participants required admission to hospital.

**Conclusions:**

For children and young people presenting to the ED with ASBD who were deemed to require oral sedative medication to assist with behavioural containment, pre‐existing mental health disorders were common. There is a need for focussed management procedures for more targeted, trauma‐focussed care for children and young people presenting to the ED with ASBD and for support and education in the pre‐hospital setting.

**Trial Registration:**

The primary study (PEAChY‐O) was registered with the Australian and New Zealand Clinical Trials Registry (ANZCTR) (ACTRN12621001236886) prior to commencement.

## Key Points

**What is already known on this topic:**

- Presentations of acute severe behavioural disturbance to emergency departments (EDs) are increasing amongst children and adolescents.
- There is limited understanding of the epidemiology of these critical events or evidence to guide their management.

**What this paper adds:**

- Of the children and adolescents who were enrolled in a randomised controlled trial of oral medication to treat acute behavioural disturbance, almost two‐thirds had previously attended the ED for management of behavioural disturbance, two‐thirds had previously reported self‐harm attempts and the most common associated medical history amongst our cohort was anxiety.
- Results will help guide the development of future studies to improve ED and community‐based management of these patients.

## 1 Introduction

The state of mental and behavioural health of children and young people was declared a national emergency in 2021 by the American Academy of Paediatrics, the American Academy of Child and Adolescent Psychiatry and the Children's Hospital Association [1]. In Australia, the most recent epidemiological report on child and adolescent mental health and wellbeing revealed that one in seven children and young people aged 4–17 years experienced a mental illness between 2013 and 2014 [2]. This rate is even higher in Europe, where a systematic review from 2024 reported that one in five children and young people have a mental health disorder [3].

As the mental and behavioural health of children and young people is vital to their cognitive, social and emotional development [1], it is imperative that the underlying causes and characteristics of acute behavioural disturbances related to mental health presentations are explored in order to improve early intervention, acute care management and service provision. Longitudinal studies highlight that most mental health disorders in adulthood begin in childhood and adolescence, such as major depressive disorders, which have an average onset of between 11 and 14 years of age [4]. There is also a link between mental health disorders beginning in childhood and poorer functional outcomes across health, education, income and social functioning [5]. Epidemiological studies will best inform care and service development to ensure present needs and concerns are met [3], to address future global socioeconomic impacts. The World Economic Forum presently identifies ill mental health as the largest contributing factor to global economic burden—more so than cardiovascular disease, cancer and diabetes [6, 7].

Health care professionals caring for children and young people experiencing a mental health or behavioural concern report that they commonly present to the emergency department (ED) for treatment [8]. In addition, the proportion of mental health related presentations are disproportionately higher than the proportion of ED presentations related to physical health concerns [9, 10]. Mental health disorders are characterised by considerable disruption to a person's emotional regulation, cognition or behaviour and can result in impaired function [3]. In children and young people, episodes of acute severe behavioural disturbance (ASBD) in the ED are increasing as, during crises, dysregulated behaviours and distress can be experienced [11, 12]. Such events are distressing and resource‐consuming in the ED setting [11] and a priority for ED‐based research [13]. To date in Australia, epidemiological data reporting on factors associated with ASBD amongst children and young people presenting to EDs are limited and have been obtained using retrospective methodologies [14].

A randomised controlled trial (RCT) of oral sedative medications was recently completed by our research team as part of a larger body of work to determine best practice management for children and young people presenting to the ED in a state of ASBD [15]. This trial compared the effectiveness of oral olanzapine and oral diazepam in achieving behavioural containment when non‐pharmacological de‐escalation attempts had been unsuccessful. No clinically important difference nor safety concerns between the study medications were found. As part of this trial, epidemiological data were recorded for all participants. This is the first study that we are aware of that captures epidemiological data from children and young people prospectively deemed to be in a state of ASBD in the ED.

The aim of the current study is to describe the epidemiology of children and young people aged nine to 17 years presenting to EDs who were deemed to require oral sedative medication to manage their ASBD.

## 2 Methods

### 2.1 Study Design

This is a secondary analysis of data collected in a randomised, controlled, open‐label, multi‐centre, phase III superiority, effectiveness, parallel drug trial in children and young people with ASBD deemed to require oral medication. The outline of the methodology of the study titled *PEAChY‐O: Pharmacological Emergency Management of Agitation in Children and Young People: A Randomised Controlled Trial of Oral Medication* is provided below with details available in a published protocol [16] and primary results paper [15].

### 2.2 Study Population

Children and young people aged between nine and 17 years deemed by the ED clinician to be in a state of ASBD based on a Sedation Assessment Tool (SAT) [17] score of ≥ +1, in whom non‐pharmacological de‐escalation attempts were unsuccessful, were considered for enrolment into the RCT. If the ED clinician decided that pharmacological management in the form of oral medication was required to manage the young person's ASBD, and no exclusion criteria were met, the young person was enrolled.

Exclusion criteria included allergies and non‐responses to either study medication; refusal of either study medication or requests for alternative therapy; as well as other safety factors outlined in the protocol paper [16].

The trial was conducted at nine Australian EDs within the Paediatric Research in Emergency Departments International Collaborative (PREDICT) Network [18], between October 2021 and November 2023.

### 2.3 Study Procedures

In the RCT, treating ED clinicians determined the level of the patient's behavioural agitation at randomisation and for primary outcome assessment using the SAT score– an ordinal seven‐point scale that rates a patient's level of agitation (*normal behaviour/not agitated = 0, mildly agitated = +1, moderately agitated = +2 to severely agitated = +3*) or sedation (*mild = −1, moderate = −2 to severe = −3*) based on speech and responsiveness [17]. Patients could be assessed for eligibility any time during their ED presentation and enrolled under a waiver of consent which was approved by the responsible ethics committee (The Royal Children's Hospital Human Research Ethics Committee Number 66478) and supported by a group of parent consumers with lived experience of caring for agitated children and young people.

Participants were enrolled using sealed, opaque randomisation envelopes at the point when the ED clinician deemed oral sedation was required to manage the patient's ASBD and facilitate ongoing care. A paper‐based case report form (CRF) (Appendix A, Figure A1) was completed prospectively by the treating clinician to capture data related to agitation and sedation levels, safety events up to 1 h post randomisation, the treating clinician's impression of the cause of ASBD, and non‐pharmacological de‐escalation attempts. All other data were collected retrospectively from participants' medical records.

In this secondary analysis, we present epidemiological data for participants enrolled in the RCT. Baseline characteristics, demographic information and intervention details were recorded for all participants up until discharge from hospital, regardless of whether the allocated study medication was ingested or not.

### 2.4 Statistical Methods

This analysis includes data from all 348 participants randomised into the RCT for whom epidemiological data were recorded in the electronic CRFs, with data combined across randomised treatment arms.

Descriptive statistics are used to summarise baseline and epidemiological characteristics of children and young people included in this secondary analysis; categorical variables are summarised using counts and percentages and continuous variables are summarised using means and standard deviations or median and interquartile range (IQR) if skewed, such as length of stay (LOS). Data were analysed using Stata Version 18.0 [19].

## 3 Results

### 3.1 Patient Characteristics

The majority of children and young people included in this study identified as cisgender female (215/348, 62%) with a mean age of 14.6 years (SD 2.2 years) (Table 1). Only a small proportion (28/348, 1%) of participants identified as non‐binary/gender diverse such as Lesbian, Gay, Bisexual, Transgender, Intersex, Queer/Questioning, Asexual plus other identity (LGBTQIA+). Fourteen percent (47/348) of participants identified as Aboriginal and/or Torres Strait Islander. Most participants were deemed moderately agitated with a SAT score of +2 at the time of randomisation (179/348, 51%). Emergency department clinicians most frequently deemed the likely cause of ASBD (prior to ascertaining a past medical history) as being due to a mental health disorder (177/348, 51%).

**TABLE 1:** Baseline characteristics of children and young people in the Emergency Department (ED) deemed to be in a state of acute severe behavioural disturbance (based on a SAT score ≥ +1) who required oral sedation for management and were enrolled in the PEAChY‐O trial.

Baseline characteristic	*N* (%) or mean (SD) *N* = 348
Age of participant (years)	Mean = 14.6 (SD = 2.2)
Participant's biological sex	
Female	215 (62%)
Participant's gender	
Female, identifies as female	191 (55%)
Male, identifies as male	129 (37%)
Non‐binary/Gender diverse	28 (8%)
Participant's ethnicity	
Australian ‐ excluding Aboriginal/Torres Strait Islander	279 (80%)
Australian ‐ Aboriginal/Torres Strait Islander	47 (14%)
Other	15 (4%)
Unknown/Not reported	7 (2%)
Agitation level (SAT score)	
+1	90 (26%)
+2	179 (51%)
+3	79 (23%)
Clinician impression of cause of ASBD^a^	
Mental health disorder	177 (51%)
Situational crisis	147 (42%)
Autism spectrum disorder	76 (22%)
Attention deficit hyperactivity disorder	59 (17%)
Substance use	51 (15%)
Intellectual disability	35 (10%)
Emotional/behavioural dysregulation	26 (8%)
Intoxication	29 (8%)
Organic illness	8 (2%)
Unknown	17 (5%)

Abbreviations: ASBD = Acute severe behavioural disturbance; SAT = Sedation and Agitation Tool; SD = standard deviation.

^a^ There are 184 individuals with more than one possible cause of acute severe behavioural disturbance (ASBD) so the total % is greater than 100.

### 3.2 Epidemiological Characteristics

The most common pre‐existing medical or mental health condition at the time of the young person's ED presentation was anxiety (122/348, 35%), followed by attention deficit hyperactivity disorder (ADHD) (115/348, 33%) and autism spectrum disorder (ASD) (112/348, 32%) (Table 2 *)*.

**TABLE 2:** Epidemiological characteristics of participants enrolled in the PEAChY‐O trial prior to arrival to ED.

Epidemiological characteristic: *N* = 348	*N* (%)
Does the participant have a past medical history?	
No	40 (12%)
Yes	299 (86%)
Unknown/Not documented	9 (3%)
Past medical history^a^	
Anxiety	122 (35%)
Attention deficit hyperactivity disorder	115 (33%)
Autism spectrum disorder	112 (32%)
Suicidal ideation	101 (29%)
Self‐harm	97 (28%)
Depression	93 (27%)
Intellectual disability	63 (18%)
Borderline personality disorder	55 (16%)
Medical or surgical history not related to mental health	49 (14%)
Post traumatic stress disorder	40 (12%)
Eating disorder	34 (10%)
Obsessive compulsive disorder	24 (7%)
Asthma	24 (7%)
Emotional/behavioural dysregulation	23 (7%)
Psychological trauma	23 (7%)
Oppositional defiance disorder	19 (6%)
Gender identity/gender dysphoria	18 (5%)
Recreational substance and/or alcohol use	14 (4%)
Schizophrenia	13 (4%)
Mental health disorder not otherwise classified	12 (3%)
Developmental disorder not specified	9 (3%)
Intentional self‐poisoning	8 (2%)
Tourette's syndrome and/or tic disorder	7 (2%)
Bipolar disorder	5 (1%)
Conduct disorder	5 (1%)
Foetal alcohol spectrum disorder	4 (1%)
Sleep disturbance or disorder	4 (1%)
Diabetes	4 (1%)
Cancer	2 (< 1%)
Other	10 (3%)
No past medical history/unknown/not documented	49 (14%)
Previous intentional self‐harm prior to current ED admission?	
No	118 (34%)
Yes^b^	212 (61%)
Unknown	18 (5%)
Type of self‐harm	
Cutting	145 (42%)
Self‐poisoning	73 (21%)
Hitting/banging head	24 (7%)
Hitting/punching/scratching/biting self/hair‐pulling	18 (5%)
Strangulation	18 (5%)
Running into traffic and/or jumping (with suicidal intent)	13 (4%)
Burning	5 (1%)
Restrictive eating	4 (1%)
Ingesting/Inserting foreign object	4 (1%)
Other	6 (2%)
Unknown	8 (2%)
Patients' residence in the week prior to arrival to ED	
Family home	234 (67%)
Group home/Residential care under Department of Health and Human Services	49 (14%)
Friends/Relatives house	24 (7%)
Foster care under Department of Health and Human Service	12 (3%)
Independent living (inclusive of hotel/motel)	11 (3%)
Emergency housing/crisis/refuge accommodation	6 (8%)
Supported accommodation/residential service/respite	5 (1%)
Homeless	3 (1%)
Public area	1 (0.3%)
Unknown	3 (1%)
Prior psychiatric care in the community/outpatient setting	
No	71 (20%)
Yes	247 (71%)
Unknown/not documented	30 (9%)
If documented prior psychiatric care (*N* = 247)	
Youth Mental Health Service	157 (64%)
Psychologist	92 (37%)
Psychiatrist	85 (34%)
General Practitioner	46 (19%)
Social worker	40 (16%)
Paediatrician	18 (7%)
Outpatient Mental Health Team	16 (6%)
School/family counsellor/therapist/youth worker	15 (6%)
Youth sexual assault service	2 (0.01%)
Aboriginal and/or Torres Strait Islander Liaison Officer	2 (0.01%)
Drug and alcohol service	2 (0.01%)
Other	8 (3%)
Previous ED presentation(s) where management of ASBD was required	
No	123 (35%)
Yes	216 (62%)
Unknown	9 (3%)
Who attended the ED with the patient?	
Parent/Legal Guardian	185 (53%)
Patient presented alone	103 (30%)
Department of Health and Human Services Worker	38 (11%)
Relative	18 (5%)
Friend	8 (2%)
Partner	5 (1%)
Teacher	2 (1%)
Aboriginal and/or Torres Strait Islander Liaison/Case Worker	1 (0.3%)
Other	2 (1%)
Unknown	3 (1%)

^a^ Research staff were encouraged to select all documented, associated medical histories so the total % is greater than 100.

^b^ Self‐harm was indicated if there was a documented account (including self‐reported) in the patient's medical record of previous self‐harm occurring any time prior to the current ED admission. This event may not have been recorded as a medical history in the hospital discharge summary notes.

Almost two‐thirds (212/348, 61%) of the participants reported previous intentional self‐harm attempts, for which the most common forms of harm were cutting (145/212, 42%) followed by self‐poisoning (73/212, 21%).

Most participants reported living in the family home prior to arrival to ED (234/348, 67%) and for more than half of the presentations (185/348, 53%), a parent/legal guardian attended the ED with the participant. However, 30% of the time (103/348), participants presented to the ED alone (either with ambulance or police staff or by themselves).

Almost three‐quarters (247/348, 71%) of participants had sought prior psychiatric care in the community or outpatient setting. The most common services engaged were Youth Mental Health Services (157/247, 64%)—integrated, specialist, state‐funded services that deliver targeted mental health early intervention and support to children and young people aged 0–18 years with complex mental health needs—followed by a psychologist (92/247, 37%) and a psychiatrist (85/247, 34%). Two‐thirds (216/348, 62%) of participants had previously attended the ED while experiencing an episode of ASBD.

Around half (178/348, 52%) of all participants arrived in an ambulance to the ED. Within this cohort, one fifth arrived with both the ambulance and police (75/348, 22%). In an additional 14% (50/348) of cases, the young person arrived with police alone (Table 3). Forty‐six percent (161/348) of participants were brought to hospital for involuntary assessment under an Australian state‐based mental health and wellbeing legislative Act.

**TABLE 3:** Epidemiological characteristics of participants enrolled in the PEAChY‐O trial during ED presentation (defined as time of triage up until time of discharge from ED).

Epidemiological characteristic	*N* (%) (or median, IQR)
Mode of arrival to ED	
Private transport	118 (34%)
Ambulance	103 (30%)
Ambulance with police escort	75 (22%)
Police alone	50 (14%)
Unknown	2 (1%)
Was the patient brought to hospital under an Australian state‐based legislative Mental Health Act or equivalent?	
No	187 (54%)
Yes	161 (46%)
Involuntary admission?^a^	
No	311 (89%)
Yes	37 (11%)
Triage category	
1	10 (3%)
2	131 (38%)
3	179 (51%)
4	26 (8%)
5	2 (1%)
Triage presenting complaint	
Psychiatric/mental health disorder	73 (21%)
Behaviour problems/behavioural disturbance	39 (11%)
Suicidal ideation	38 (11%)
Mental health problem	35 (10%)
Suicidal attempt	31 (9%)
Aggression/agitation	26 (8%)
Self‐harm	26 (8%)
Drug Ingestion/overdose/intoxication	25 (7%)
Situational crisis/psychosocial presentation	15 (4%)
Eating disorder/decreased oral intake	9 (3%)
Destructive behaviour	7 (2%)
Miscellaneous	5 (1%)
Foreign body	1 (0.3%)
Laceration	1 (0.3%)
Other presenting triage complaint not related to mental health disorder	17 (5%)
Was the patient admitted to hospital?	
No	250 (72%)
Yes	98 (28%)
Length of stay (LOS) (hours) in the ED from time of triage	Median = 5.7 (IQR = 3.9, 10.2)
LOS (hours) in the hospital from time of triage	Median = 7.6 (IQR = 4.2, 26.8)
Disposition of patient after leaving ED	
Discharged home	171 (49%)
Admitted to mental health unit	62 (18%)
Group home under Welfare Services	24 (7%)
Admitted to paediatric/general medical unit or similar	19 (6%)
Transferred to another mental health facility	13 (4%)
Discharged to Supported accommodation/residential service	12 (3%)
Admitted to adolescent ward or similar	12 (3%)
Foster care under Welfare Services	9 (3%)
Discharged into care of relative (outside the home)	8 (2%)
Discharged into care of non‐legal guardian/s	5 (1%)
Discharged to Emergency housing/Crisis/Refuge accommodation	3 (1%)
Police custody	3 (1%)
Transferred to another adolescent facility	2 (1%)
Absconded/Left at own risk	2 (1%)
Juvenile justice	1 (0.3%)
Transferred to another paediatric/general medical unit	1 (0.3%)
Unknown	1 (0.3%)

^a^ Involuntary admission utilising an Assessment Order (AO): enables an authorised mental health practitioner or doctor to examine a person to determine if they have a mental illness and require compulsory treatment.

Forty‐one percent (Australian Triage Scale (ATS) 1: 10/348, 3%; ATS 2: 131/348, 38%) of participants were allocated a triage category 1 or 2, indicating requirement of treatment immediately or within 10 min of presenting to the ED [20]. The most common triage presenting complaint was described as a ‘psychiatric/mental health disorder’ (73/348, 21%). The median length of stay (LOS) in the ED was 5.7 h (IQR 3.9–10.2 h) from the time of triage and 28% (98/348) of participants were admitted to hospital.

Participants were discharged from the ED to home half of the time (171/348, 49%), whilst 18% (62/348) were admitted to a child and adolescent mental health unit and 14% were discharged into supported, child protection or crisis care facilities under welfare services which consisted of a group home in 7% (24/348), supported accommodation/residential service in 3% (12/348), foster care in 3% (9/348) and emergency housing/crisis/refugee accommodation in 1% (3/348) of participants. It is presumed that the participant's disposition was to the same residence/facility that they came from prior to arrival to the ED (except in instances where their residence in the week prior to presenting to the ED was documented as ‘homeless, public area or unknown’).

## 4 Discussion

This study provides a prospective description of the demographic and epidemiological characteristics of children and young people presenting to Australian EDs in a state of ASBD requiring oral medication management. Prior investigations in this population have largely relied on retrospective chart reviews, which are constrained by the accuracy and completeness of medical records [14]. By contrast, the present data were collected prospectively, offering a more comprehensive and reliable characterisation of this cohort.

There is a paucity of data describing factors related specifically to ASBD events amongst children and young people. This absence of evidence can impede efforts to understand and subsequently adequately prevent behavioural disturbance in this population which affects the establishment of best management recommendations [4].

Several national retrospective cohort studies and a systematic review on children and young people presenting to the ED with a mental health disorder, some specifically involving an ASBD event, also support our findings of a female preponderance and a mean age in the mid‐teens [10, 12, 14, 21]. The reason that female children and young people are more likely to present to EDs with a mental health condition or with ASBD is unknown. A large‐scale survey of Australian children and young people with mental health disorders revealed that male children and young people are overall more likely to experience a mental health disorder [2]. Possible explanations for this discrepancy may be that female children and young people are less likely to externalise their discomfort or disclose their concerns. Self‐harm in the Australian population is twice as common amongst female children and young people as males [2]. As such, the higher rate of females presenting to the ED in a state of ASBD may be due to the need for treatment of self‐harm injuries.

There is a strong association between self‐harm and anxiety disorders or depression amongst children and young people [22]. Concerningly, suicide is the second most common cause of death worldwide after road traffic accidents amongst 10–24 year‐olds and non‐fatal self‐harm is the strongest risk factor associated with subsequent suicide [22]. Furthermore, attempted suicide‐related ED visits experienced the greatest increase amongst all paediatric ED presentations from 2011 to 2020 [9]. These growing trends indicate the importance of more targeted primary care screening, early intervention and support (including family, school and community engagement and education) [3, 6, 23] of children and young people diagnosed with anxiety and depression. Understanding the associated risk factors for and progression of mental health disorders may also result in net economic savings. An Australian study found that investment in prevention and early treatment services for anxiety and depression could result in short‐term savings of up to $90 million [24]. Services included e‐Health mental health interventions (e.g., MoodGYM), school‐based anti‐bullying, psychological programs and parenting interventions [25]. Another smaller, community suicide‐prevention intervention model (Live4Life) in rural Australia found that increased mental health knowledge and help‐seeking initiatives resulted in decreased mental health stigma and reduced suicidal ideation and attempts in children and young people [23].

We found that anxiety is the most common mental health disorder amongst children and young people presenting to EDs in a state of ASBD. This finding is reflected in the literature [3]. It is not to say that all children and young people with anxiety will experience behavioural crises and require management in the ED. It is worth acknowledging, though, that in the acute care environment, children and young people with a history of anxiety may experience heightened feelings of anxiety, which may evolve into behavioural disturbance.

Similar to our cohort, previous literature has demonstrated that children and young people with ADHD more commonly experience ASBD [26]. Children and young people with ADHD experience difficulty with emotional regulation, impulsivity, and poor executive functioning, which may lead to episodes of ASBD pre‐arrival to the ED and may be exacerbated in the stressful ED environment [26]. Given that an ED presentation can be triggering to this population and distressing and disruptive for the entire family unit, focused community support services for parents/guardians of children and young people diagnosed with anxiety and/or a neurodevelopmental disorder may be beneficial. There is emerging evidence that early diagnosis and support, to build family resilience, may reduce behavioural and mental health problems in young people with these mental health disorders [27].

Access limitations to primary care mental health treatment services such as youth mental health services, psychologists, or psychiatrists result from financial barriers, ongoing stigma, and a lack of engagement [6]. This may lead to a ‘domino effect’ of social and economic burdens due to associated healthcare, education, child protection, and justice system costs and demands [25]. These impacts are experienced first‐hand by emergency responders, for example, paramedics, police, and ED staff, when crisis point is reached and children and young people and/or their family members seek or require urgent care, such as is the case during an episode of ASBD. However, in the current study, we found that a large proportion of participants had sought prior psychiatric care in the community or outpatient setting but still presented to the ED in a state of ASBD. This again raises the question as to whether current approaches to acute crisis care need to shift focus from being young person centric and reactive to the provision of preventative care in the form of targeted, trauma‐focussed recognition of possible triggers, education, and support for the wider community (i.e., family, school, and workplaces).

Our study also reports an over‐representation of Aboriginal and/or Torres Strait Islander Australian children and young people presenting to the ED in a state of ASBD for whom oral sedation was required. The most recent estimates from the Australian Institute of Health and Welfare report that 4% of the total Australian population identifies as Aboriginal and/or Torres Strait Islander [28]. The percentage of participants identifying as Aboriginal and/or Torres Strait Islander in the regions that the study sites were situated ranged from 1% to 5% [28]. However, 14% of the study participants identified as Aboriginal and/or Torres Strait Islander. This over‐representation of mental health disorders amongst Aboriginal and/or Torres Strait Islander children and young people is purported to be due to the impacts of intergenerational trauma linked to the legacy of colonisation and, subsequently, a result of cultural genocide, disempowerment, racism and disadvantage [29, 30]. More focussed epidemiological research is required to determine current associations between mental health disorders and ASBD, as well as care needs in the First Nation population to support recommendations in emerging national frameworks and policies.

Although we are able to report novel findings, a number of limitations are identified. This analysis is limited to participants enrolled in the PEAChY‐O RCT. Therefore, due to trial exclusion criteria, data presented here may not be representative or generalisable to all children and young people who present to the ED for ASBD management. Regardless, data on associated patterns of presentations and health service use can assist healthcare professionals or policy makers in identifying and remediating gaps. We also did not directly measure causality related to ASBD events. Further qualitative research is required to determine triggers of ASBD in children and young people prior to and on presentation to acute care settings. The study team recommends that future studies should include data related to contributing factors such as department status (i.e., staffing, waiting times, noise and utilisation of quiet spaces or low stimulus rooms) to specifically improve care for patients experiencing ASBD in the ED or to mitigate behavioural escalations beforehand. The epidemiological data reported in this study provides a steppingstone for future research.

## 5 Conclusion

This study is the first to report epidemiological data for children and young people presenting to EDs in a state of ASBD and who were deemed to require oral sedative medication. Factors associated with ASBD presentations are multifactorial. Participants frequently re‐presented; the most common past medical history for this cohort was anxiety and a high proportion reported previous self‐harm. Further epidemiological research should target triggers and the impact of decisions in the home and pre‐hospital setting to better understand the impact of the underlying conditions on the causes and differential management of ASBD.

## Author Contributions

E.M.B., K.K. and F.E.B.: conceptualised and designed the RCT, designed the data collection instruments, collected data, drafted and critically reviewed and revised the initial manuscript. E.M.B. and K.K.: first drafts of the resulting paper. K.K.: submitting author. C.J.S. and K.L.: statistical analyses, interpretation and revision of first drafts. S.S.C. and F.E.B.: acquired funding. A.K., D.S., M.L.B., S.G., S.J., D.T., M.G., C.L.W., C.P., N.P., G.N., A.D. and J.C.K.: Revision of the resulting paper for content, including medical writing for content; major role in the acquisition of data from their sites; study concept and design; analysis and interpretation of data. All authors agree to be accountable for the accuracy and integrity of the work. Guarantor: E.M.B. is the guarantor and accepts full responsibility for the overall content, the conduct of the study, and controlled the decision to publish.

## Funding

The study was funded by a grant from the National Health and Medical Research Council (NHMRC) Medical Research Future Fund (MRFF) Million Minds Mental Health Research Mission Grant (GNT1179137), Canberra, Australia and the Perth Children's Hospital Foundation project grant (#9670), Perth, Australia. The PREDICT research network was part funded by an NHMRC Centre of Research Excellence grant (GNT1058560), Canberra, Australia, the Murdoch Children's Research Institute, Melbourne, Australia, and the Victorian Government's Operational Infrastructure Support program. F.E.B.'s time was part funded by a grant from the Royal Children's Hospital Foundation, Melbourne, Victoria, Australia and an NHMRC Investigator grant (GNT2017605). K.L.'s time was part funded by an NHMRC Investigator grant (GNT2017498). E.M.B.'s time was part funded by an NHMRC postgraduate scholarship (GNT 2005279). The funders didn't influence the results/outcomes of the study despite author affiliations with the funders. The NHMRC had no role in the design and conduct of the study.

## Disclosure

AI technology was not used in the course of this research.

## Ethics Statement

Ethical approval for the primary study (PEAChY‐O) was obtained from The Royal Children's Hospital (RCH), Melbourne Human Research Ethics Committee (HREC). The study operated under a waiver of consent.

## Conflicts of Interest

The authors declare no conflicts of interest.

## Supporting information


**Data S1:** jpc70499‐sup‐0001‐STROBE‐Checklist.docx. STROBE Statement—checklist of items that should be included in reports of observational studies.

## Figures and Tables

**FIGURE A1:**
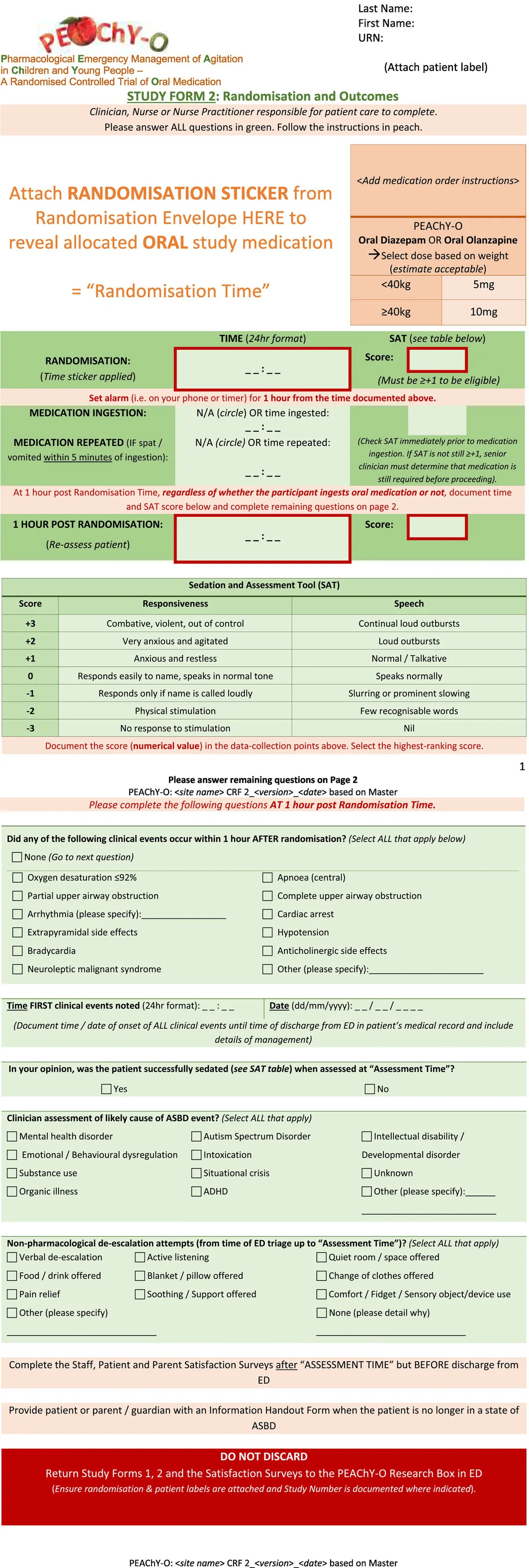
Case Report Form (CRF) completed by ED clinicians from time of triage up until 1 h post randomisation time.

## Data Availability

The PEAChY‐O trial data are held at the Murdoch Children's Research Institute in Melbourne, Australia. Requests for data can be made at any time and can be initiated by email to elyssia.bourke@mcri.edu.au. Requests will be considered within existing administrative, ethical and regulatory constraints and ongoing research by the study team. Deidentified individual participant data will not be made available.
